# Antimicrobial and antioxidant activity of kaempferol rhamnoside derivatives from *Bryophyllum pinnatum*

**DOI:** 10.1186/1756-0500-5-158

**Published:** 2012-03-20

**Authors:** Simplice Joel Ndendoung Tatsimo, Jean de Dieu Tamokou, Léopold Havyarimana, Dezső Csupor, Peter Forgo, Judit Hohmann, Jules-Roger Kuiate, Pierre Tane

**Affiliations:** 1 Department of Chemistry, Higher Teachers' Training College, University of Maroua, P.O. Box 55, Maroua, Cameroon; 2 Department of Biochemistry, Faculty of Science, University of Dschang, P.O. Box 67, Dschang, Cameroon; 3 Department of Chemistry, Faculty of Science, University of Burundi, P.O. Box 2700, Bujumbura, Burundi; 4 Department of Pharmacognosy, University of Szeged, Eötvös u. 6, H-6720 Szeged, Hungary; 5 Department of Chemistry, University of Dschang, PO Box 67, Dschang, Cameroon

**Keywords:** *Bryophyllum pinnatum*, Crassulaceae, Kaempferol rhamnosides, Antimicrobial, Antioxidant, Minimum inhibitory concentration

## Abstract

**Background:**

*Bryophyllum pinnatum *(Lank.) Oken (Crassulaceae) is a perennial succulent herb widely used in traditional medicine to treat many ailments. Its wide range of uses in folk medicine justifies its being called "*life plant*" or "*resurrection plant*", prompting researchers' interest. We describe here the isolation and structure elucidation of antimicrobial and/or antioxidant components from the EtOAc extract of *B. pinnatum*.

**Results:**

The methanol extract displayed both antimicrobial activities with minimum inhibitory concentration (MIC) values ranging from 32 to 512 μg/ml and antioxidant property with an IC_50 _value of 52.48 μg/ml. Its partition enhanced the antimicrobial activity in EtOAc extract (MIC = 16-128 μg/ml) and reduced it in hexane extract (MIC = 256-1024 μg/ml). In addition, this process reduced the antioxidant activity in EtOAc and hexane extracts with IC_50 _values of 78.11 and 90.04 μg/ml respectively. Fractionation of EtOAc extract gave seven kaempferol rhamnosides, including; kaempferitrin (**1**), kaempferol 3-*O*-α-L-(2-acetyl)rhamnopyranoside-7-*O*-α-L-rhamnopyranoside (**2**), kaempferol 3-*O*-α-L-(3-acetyl)rhamnopyranoside-7-*O*-α-L-rhamnopyranoside (**3**), kaempferol 3-*O*-α-L-(4-acetyl)rhamnopyranoside-7-*O*-α-L-rhamnopyranoside (**4**), kaempferol 3-*O*-α-D- glucopyranoside-7-*O*-α-L-rhamnopyranoside (**5**), afzelin (**6**) and α-rhamnoisorobin (**7**). All these compounds, except **6 **were isolated from this plant for the first time. Compound **7 **was the most active, with MIC values ranging from 1 to 2 μg/ml and its antioxidant activity (IC_50 _= 0.71 μg/ml) was higher than that of the reference drug (IC_50 _= 0.96 μg/ml).

**Conclusion:**

These findings demonstrate that *Bryophyllum pinnatum *and some of its isolated compounds have interesting antimicrobial and antioxidant properties, and therefore confirming the traditional use of *B. pinnatum *in the treatment of infectious and free radical damages.

## Background

*Bryophyllum pinnatum *(Lank.) Oken, *syn*. *B. calucinum *or *Kalanchoe pinnata *(Crassulaceae) is a perennial succulent herb which grows in Africa and Asia [1]. An ethnobotanical survey of plants used in the treatment of infectious diseases in Mbouda subdivision (Cameroon) indicated that *B. pinnatum *was one of the most used medicinal plants in the area. Therefore information collected directly from traditional healers and herbal sellers in this area indicated that, leaves or the whole plant are used as analgesic and to treat blennorrhoea, syphilis, jaundice, candidiasis, dysmenorrhoea, external ulcers, burns and convulsions. *B. pinnatum *is also used elsewhere for treatment of ear infections, cough and dysentery [2]. This wide range of traditional uses justifies its being called "*life plant*", "*resurrection plant*" or "*goodluck*" [3,4]. Previous phytochemical studies revealed the presence of terpenoids [1], cytotoxic bufadienolides [5] and antileishmanial flavonoids [6] in this plant. In addition, 60% methanolic extract of leaves of *B. pinnatum *have shown antimicrobial activity [2], while water extracts have shown anti-ulcer, antinociceptive, anti-inflammatory, antidiabetic, neurosedative and muscle relaxant activities [3,4,7], and ethanolic extract have exhibited hepatoprotective activity [8]. In the course of our search for bioactive components from Cameroonian medicinal plants, phytochemical and biological investigations of *B. pinnatum *were carried out. We describe here the isolation and structure elucidation, antimicrobial and antioxidant properties of seven kaempferol rhamnosides (**1**-**7**) from *B. pinnatum *which may account for some of the ethnomedicinal uses of leaves or the whole plant.

## Results

### Chemical analysis

**Figure 1:**
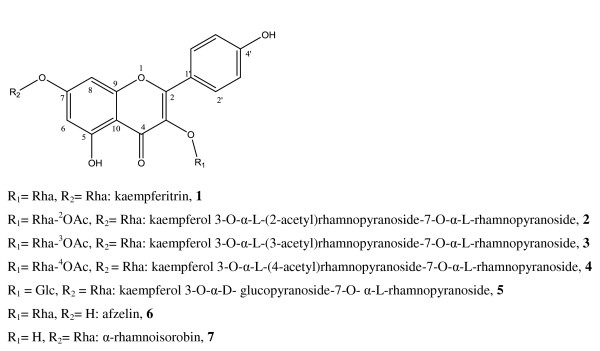
**Chemical structures of compounds isolated from *B. pinnatum***.

The whole plant of *B pinnatum *was dried between 36-38°C during two weeks. This raw material (1.50 Kg) was extracted with MeOH and the MeOH extract was partitioned with hexane and ethyl acetate. The resulting extracts underwent antimicrobial (antibacterial and antifungal) and antioxidant assays revealing MICs values from 16-1024 μg/ml and IC_50 _values from 52.48-90.04 μg/ml. The antimicrobial activity was found to be more concentrated in EtOAc extract and diluted in hexane extract. The EtOAc extract was then subjected to column chromatography to yield seven kaempferol derivatives: kaempferitrin (435.10 mg) (**1**) [9], kaempferol 3-*O*-α-L-(2-acetyl)rhamnopyranoside-7-*O*-α-L-rhamnopyranoside (10.10 mg) (**2**) [10], kaempferol 3-*O*-α-L-(3-acetyl)rhamnopyranoside-7-*O*-α-L-rhamnopyranoside (38.40 mg) (**3**) [10], kaempferol 3-*O*-α-L-(4-acetyl)rhamnopyranoside-7-*O*-α-L-rhamnopyranoside (25.10 mg) (**4**) [11], kaempferol 3-*O*-α-D- glucopyranoside-7-*O*- α-L-rhamnopyranoside (40,90 mg) (**5**) [12], afzelin (34.60 mg) (**6**) [9] and α-rhamnoisorobin (5.0 mg) (**7**) [9] (Figure 1). These compounds except afzelin (**6**) were isolated for the first time in this species.

### Antimicrobial activity

The MeOH, EtOAc and hexane extracts as well as isolated compounds were tested for their antimicrobial activity and the results obtained are presented in Table 1. The crude extracts have shown both antibacterial and antifungal activities, on the set of germs tested with MIC values ranging from 16 to 1024 μg/ml. The EtOAc extract was more active (MIC = 16-128 μg/ml) than the MeOH extract (MIC = 32-512 μg/ml) and the hexane extract (MIC = 128-1024 μg/ml), meaning that the active principles might be more concentrated in the EtOAc extract. Compound **7 **isolated from the EtOAc extract was more active (MIC = 1-2 μg/ml) than all the other tested compounds. This is followed in decreasing order by compounds **5**, **6**, **1**, **3 **and **4**. The antimicrobial activity of compound **7 **was in some cases equal to those of the positive controls (ciprofloxacin and nystatin). Nevertheless, the antibacterial activity of this compound (MIC = 1 μg/ml) was higher than that of the positive control ciprofloxacin (MIC = 2 μg/ml) against *Pseudomonas aeruginosa*.

**Table 1:** Antimicrobial activity (in μg/ml) of crude extracts of B. *pinnatu m *and isolates from EtOAc extract

*Test samples (extracts and isolates 1-7)*	*Inhibition parameters*	*Staphylococcus aureus*	*Pseudomonas aeruginosa*	*Salmonella typhi*	*Candida albicans*	*Candida parapsilosis*	*Cryptococcus neoformans*
MeOH extract	MIC	256	512	64	64	32	32
	
	MMC	256	512	128	64	32	64
	
	MMC/MIC	1	1	2	1	1	2

EtOAc extract	MIC	64	128	16	32	16	16
	
	MMC	64	256	16	32	32	16
	
	MMC/MIC	1	2	1	1	2	1

*n-*hexane extract	MIC	512	1024	256	256	128	1024
	
	MMC	512	1024	256	256	128	1024
	
	MMC/MIC	1	1	1	1	1	1

**1**	MIC	32	32	32	32	16	16
	
	MMC	32	64	32	64	16	16
	
	MMC/MIC	1	2	1	2	1	1

**4**	MIC	64	128	32	64	32	32
	
	MMC	64	128	32	128	32	32
	
	MMC/MIC	1	1	1	2	1	1

**6**	MIC	8	16	2	16	4	4
	
	MMC	8	16	2	32	4	4
	
	MMC/MIC	1	1	1	2	1	1

**5**	MIC	4	4	1	8	2	2
	
	MMC	8	4	2	8	2	4
	
	MMC/MIC	2	1	2	1	1	2

**7**	MIC	2	1	1	1	2	2
	MMC	2	2	1	1	2	2
	
	MMC/MIC	1	2	1	1	1	1

**3**	MIC	256	128	64	32	16	16
	
	MMC	256	128	64	32	32	16
	
	MMC/MIC	1	1	1	1	2	1

Ciprofloxacin	MIC	2	2	0.5	/	/	/
	
	MMC	2	2	1	/	/	/
	
	MMC/MIC	1	1	2	/	/	/

Nystatin	MIC	/	/	/	2	1	0.5
	
	MMC	/	/	/	2	1	0.5
	
	MMC/MIC	/	/	/	1	1	1

/: not determined

### Antioxidant activity

The results of the antioxidant activities of the MeOH, EtOAc and hexane extracts as well as their isolated compounds are presented in Table 2. It appeared that the MeOH extract (IC_50 _= 52.48 μg/ml) was more active than the EtOAc extract (IC_50 _= 78.11 μg/ml) and hexane extract (IC_50 _= 90.04 μg/ml). The antioxidant activity of compound **7 **(IC_50 _= 0.71 μg/ml) was higher than that of the reference drug (IC_50 _= 0.96 μg/ml). No biological test was done with compound **2 **since it was obtained as a mixture with compound **3**.

**Table 2:** Inhibition concentrations of the test samples scavenging 50% of DPPH radical (IC_50_)

Test samples (extracts and isolates 1-7 from EtOAc extract)	IC_50 _(μg/ml)
MeOH extract	52.48 ± 0.19^a^

EtOAc extract	78.11 ± 0.61^b^

*n*-hexane extract	90.04 ± 1.26^c^

**1**	8.73 ± 0.51^d^

**4**	273.81 ± 0.43^e^

**3**	487.99 ± 0.81^f^

**6**	6.44 ± 0.74^g^

**5**	2.28 ± 0.12^h^

**7**	0.71 ± 0.09^i^

Vitamin C	0.96 ± 0.14^j^

Values are expressed as mean ± SD. In the same column, values affected by the different superscript letters (a-j) are significantly different *p *< 0.05.

## Discussion

The findings of the present study showed differences between the antimicrobial activities of crude MeOH extract and extracts from partition. This suggests that *B. pinnatum *contains several antifungal and antibacterial principles with different polarities as shown by the phytochemical study. The partition of the MeOH extract enhanced its antimicrobial activity in EtOAc fraction, and reduced that of hexane fraction. This indicates that the active principles might be more concentrated in EtOAc fraction and more diluted in hexane fraction. All the isolated compounds showed antimicrobial activities on at least one microorganism. Such a finding supports the traditional use of this plant in the treatment of infectious diseases. The result of the antimicrobial activity of MeOH extract from the whole plant of *B. Pinnatum *corroborates that of Akinpelu [2].

The antimicrobial activities varied with the bacterial and fungal species. These variations may be due to genetic differences between the microorganisms. A keen look at the results of MIC and minimum microbicidal concentration (MMC) (Table 1), showed that the MIC values obtained are in most cases equal to the MMC values on the corresponding (sensitive) microorganisms, confirming the microbicidal effects of the tried samples [13]. This is interesting in view of the perspective of developing new antibacterial drugs from natural products.

The antimicrobial activity was more concentrated in EtOAc fraction. In contrast, the antioxidant activity is more concentrated in MeOH extract. This indicates that partition of the crude extract did not enhance the antioxidant activity of its fractions. Flavonoid compounds such as compounds **1**-**7 **are known to be potential antioxidant due to their ability to scavenge free radicals and active oxygen species such as singlet oxygen, superoxide anion radical and hydroxyl radicals [14,15]. The antimicrobial activity of flavonoids (compounds **1**-**7) **might be due to their ability to complex with bacteria cell wall and therefore, inhibiting the microbial growth [16]. The presence of these compounds could explain the antioxidant activity found in the crude extract.

Compounds **1**, **3**-**7 **displayed both antibacterial and antifungal activities. However, this is the first report concerning the antimicrobial and antioxidant activities of these compounds.

The overall results of this study can be considered as very promising in the perspective of new drugs discovery from plant sources, when considering the medical importance of tested microorganisms. *Staphylococcus aureus *is a major cause of community and hospital-associated infection with an estimated mortality of around 7-10% [17]. About 77% of immune-deficient patients' death is attributed to microscopic fungi, such as *Candida *species and *Cryptococcus neoformans *[18]. Also, *Candida albicans *has been reported to account for 50-70% cases of invasive candidiasis [19]. Alarmingly, the incidence of nosocomial candidemia has risen sharply in the last decade [20]. All this has resulted in severe consequences including increased cost of medicines and mortality of patients. Typhoid fever caused by *Salmonella typhi *continues to be a serious public health problem in developing countries in general and in Sub-Saharan Africa in particular [17]. Generally, these pathogens were found to be sensitive to extracts and isolated compounds.

## Conclusion

These findings demonstrated that methanol and ethyl acetate extracts of *B. pinnatum *and the isolated compounds exhibited interesting antimicrobial and antioxidant properties, justifying the traditional uses of the plant in the treatment of infectious diseases and free radical damages. However, further pharmacological and toxicological studies need to be done in order to confirm or infirm this hypothesis.

## Methods

### Plant material

The whole plant of *B. pinnatum *was collected in Mbouda subdivision, West region of Cameroon in August 2009. It was identified by Mr. Nana at the National Herbarium, Yaoundé where a voucher specimen 33394 HNC describing the plant is deposited.

### Extraction and isolation

Dried and ground whole plant of *B. pinnatum *[1] (1.50 Kg) was extracted by percolation with methanol (10 L) at room temperature. Filtration and evaporation of solvent under reduced pressure gave a brown residue (148 g). This extract was successively partitioned with *n*-hexane and ethyl acetate to yield 38.03 and 34.03 g of extracts respectively [21]. All extracts were kept in the refrigerator at around 4°C.

The EtOAc extract (34.03 g) was subjected to silica gel column chromatography (6 × 30 cm, 300 g) eluted with a gradient system of hexane-EtOAc-MeOH gradients. 182 fractions of 200 mL each were collected and combined according to TLC profile into nine main fractions: A (fractions 1-45), B (fractions 46-70), C (fractions 71-92), D (fractions 93-109), E (fractions 110-129), F (fractions 130-140), G (fractions 141-154), H (fractions 155-170), I (fractions 171-182). G (8.91 g) was loaded on a silica gel column eluted with CH_2_Cl_2_-MeOH gradients and 200 fractions of 13 ml each were collected. Subfractions 114-140 obtained with CH_2_Cl_2_-MeOH (87.5-12.5, 200 mL; 85-15, 200 mL) were again subjected to silica gel column chromatography eluted with a gradient system of EtOAc-MeOH-H_2_O to yield 50 fractions of 13 mL each. Subfractions 8-11 obtained with EtOAc-MeOH-H_2_O (98-02-0.1) were purified on a sephadex LH-20 column eluted with MeOH to give afzelin (**6**) as yellow crystal; and α-rhamnoisorobin (**7**) as amorphous yellowish pate. Subfractions 17-32 obtained with EtOAc-MeOH-H_2_O (98-02-0.1 and 96-04-0.1) were loaded on vaccum RP-18 column eluted with H_2_O-MeOH gradients (60-40 to 0-100) and fractions obtained were loaded on preparative RP-TLC to yield kaempferol 3-*O*-α-L-(3-acetyl)rhamnopyranoside-7-*O*-α-L-rhamnopyranoside (**3**), kaempferol 3-*O*-α-L-(4-acetyl)rhamnopyranoside-7-*O*-α-L-rhamnopyranoside (**4**) and a mixture of kaempferol 3-*O*-α-L-(2-acetyl)rhamnopyranoside-7-*O*-α-L-rhamnopyranoside (**2**) and compound **3**. Subfractions 141-155 from G, obtained with CH_2_Cl_2_-MeOH (81.5-17.5; 200 mL) were purified on a sephadex LH-20 column eluted with MeOH to yield yellow crystal of kaempferitrin (**1**). I (821 mg) was loaded on vaccum RP-column eluted with H_2_O-MeOH gradients (60-40 to 0-100) and 67 fractions of 15 mL each were collected. Subfractions 30-45 obtained with H_2_O-MeOH (85-15) were again subjected to RP-column eluted with H_2_O-MeOH (60-0 to 40-60) and fractions were purified on MeOH sephadex column and an isocratic H_2_O-MeOH (80-20) RP-column to yield 3-*O*-α-D-glucopyranoside-7-*O*-α-L-rhamnopyranoside (**5**). The chemical structures of the isolated compounds are shown in Figure 1.

### General procedure

NMR spectra were measured on Bruker Avance DRX 500 and Brucker Ultrashield Plus 600 spectrometers at 500 and 600 MHz for ^1^H, and 125 and 150 MHz for ^13 ^C NMR, with TMS as internal standard; chemical shifts are given in δ values (ppm). MS analyses were performed using a QTOF Premier (Waters, Milford, MA) equipped with a nanoelectrospray ionization source. The instrument was operated in positive ion mode, performing full-scan analysis over the *m*/*z *range 50-1990 at 1 spectra/s. Column chromatography was run on Merck silica gel 60 (0.063-0.200 mm), Lichoprep RP-18 (40-63 μm) and Sephadex LH-20 while TLC was carried out on silica gel GF_254 _pre-coated plates with detection accomplished by visualizing with a UV lamp at 254 and 365 nm, followed by spraying with vanillin and then heating at 100°C.

### Biological tests

#### Antimicrobial activity

##### Microorganisms

The microorganisms used in this study consisted of three bacteria (*Staphylococcus aureus *ATCC25922, *Pseudomonas aeruginosa *ATCC27853, *Salmonella typhi *ATCC6539) and two *Candida *species (*Candida albicans *ATCC9002 and *Candida parapsilosis *ATCC22019), all of which are reference strains obtained from the American Type Culture Collection. Also, included was one strain of *Cryptococcus neoformans *IP95026 obtained from the Pasteur Institute (IP, Paris-France). The bacterial and yeast strains were grown at 35°C and maintained on nutrient agar (NA, Conda, Madrid, Spain) and Sabouraud Dextrose Agar (SDA, Conda) slants, respectively.

##### Determination of Minimum Inhibitory Concentration (MIC) and Minimum Microbicidal Concentration (MMC)

MICs were determined by broth micro dilution method as described by Nyaa et al. in 2009 with slight modifications [22]. The test samples were first of all dissolved in dimethylsulfoxide (DMSO). The solution obtained was then added to Mueller Hinton Broth (MHB) for bacteria or Sabouraud Dextrose Broth (SDB) for yeasts to give a final concentration of 2048 μg/ml. This was serially diluted two fold to obtain concentration ranges of 0.50 to 2048 μg/ml. Each concentration (100 μl) was added in each well (96-wells microplate) containing 95 μl of MHB or SDB and 5 μl of inoculum for final concentrations varying from 0.25 to 1024 μg/ml. The inoculum was standardized at 1.50 × 10^6 ^CFU/ml by adjusting the optical density to 0.10 at 600 nm JENWAY 6105 UV/Vis spectrophotometer. The final concentration of DMSO in each well was less than 1% (preliminary analyses with 1% (v/v) DMSO do not inhibit the growth of the test organisms). The negative control well consisted of 195 μl of appropriate medium (MHB for bacteria and SDB for yeasts) and 5 μl of the standard inoculum. The plates were covered with the sterile lid, then agitated to mix the contents of the wells using a plate shaker and incubated at 35°C for 24 h (for bacteria) or for 48 h (for yeasts). The assay was repeated thrice. The MICs of samples were determined by adding 50 μl of a 0.2 mg/ml *p*-iodonitrotetrazolium violet solution followed by incubation at 35°C for 30 min. Viable micro-organisms reduced the yellow dye to a pink color. MICs were defined as the lowest sample concentrations that prevented this change in color indicating a complete inhibition of microbial growth.

For the determination of MMCs, a portion of liquid (5 μl) from each well that showed no growth of microorganism was placed on Mueller Hinton Agar or Sabouraud Dextrose Agar and incubated at 35°C for 24 h (for bacteria) or 35°C for 48 h (for yeasts). The lowest concentrations that yielded no growth after this sub-culturing were taken as the MMCs [22]. Ciprofloxacin (Sigma-Aldrich, Steinheim, Germany) and nystatin (Merck, Darmstadt, Germany) for bacteria and yeasts, respectively, were used as positive controls.

### Antioxidant assay

The antioxidant activities of methanol, EtOAc and hexane extracts, and isolated compounds were evaluated by 2,2-diphenyl-1-picryl-hydrazyl-hydrate (DPPH) radical scavenging method as described by Mensor et al. in 2001 with slight modifications [23]. The test samples, initially dissolved in DMSO (SIGMA) were mixed with a 20 mg/l of 2,2-diphenyl-1-picryl-hydrazyl-hydrate (DPPH) methanol solution, to give final concentrations of 10, 50, 100, 500 and 1000 μg/ml. After 30 min at room temperature, the absorbance values were measured at 517 nm and converted into percentage of antioxidant activity as follows:

% Inhibition = (Absorbance of control - Absorbance of test sample) × 100/Absorbance of control

L-ascorbic acid was used as a standard control. The inhibition ratio was converted in probits. The probit values were plotted against the logarithmic values of concentrations of the test samples and a linear regression curve was established in order to determined the IC_50 _(μg/ml) values, which is the amount of sample necessary to decrease by 50% the absorbance of DPPH. All the analyses were carried out in triplicate and the results were expressed as the mean ± standard deviation (SD) and compared using Waller-Duncan test. A value of *p *< 0.05 was considered statistically significant.

## Competing interests

The authors declare that they have no competing interests.

## Authors' contributions

JSNT designated the study, did the isolation and structure elucidation part with the help of DC, under the supervision of PT and JH. PF measured the NMR data of all the isolates. JDT and JRK did the biological part and participated in the preparation of the manuscript. LH helped in plant selection, study designation, manuscript writing and editing. All authors read and approved the final manuscript.
